# Long non-coding RNA RGMB-AS1 represses nasopharyngeal carcinoma progression via binding to forkhead box A1

**DOI:** 10.1080/21655979.2022.2039495

**Published:** 2022-02-19

**Authors:** Tian Zhang, Ying Jin, Xiangmei Luo

**Affiliations:** a Department of ENT and HN Surgery, Affiliated Hospital of Guizhou Medical University, Guiyang, PR China; b Department of ENT and HN Surgery, Central People’s Hospital of ZhanJiang, ZhanJiang, PR China

**Keywords:** Nasopharyngeal carcinoma, lncRNA RGMB-AS1, FOXA1, proliferation, EMT

## Abstract

Long non-coding RNA RGMB-AS1 (RGMB antisense RNA 1) plays a crucial role in tumor progression. However, its underlying mechanism in nasopharyngeal carcinoma (NPC) remains unclear. In this study, we analyzed the clinical significance of lncRNA RGMB-AS1 as a possible potential marker in NPC, and investigated the effect and mechanism of lncRNA RGMB-AS1 on proliferation, migration and epithelial mesenchymal transformation (EMT) of NPC by directly binding Forkhead box A1 (FOXA1) in *vitro* and in *vivo*. In conclusion, LncRNA RGMB-AS1 inhibits malignant behaviors and EMT by regulating FOXA1, and lncRNA RGMB-AS1 may be an important indicator of clinical prognosis.

## Graphical Abstract

**Figure:**
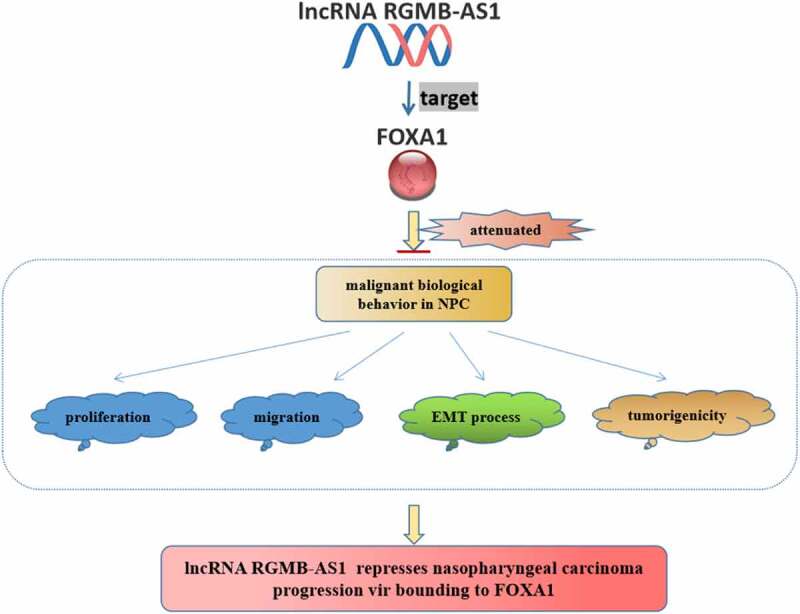

## 1 Introduction

Nasopharyngeal carcinoma (NPC) is one of the most common tumors in the head and neck, mainly concentrated in Southeast Asia, and it is the most common invasive squamous cell carcinoma [1]. Early NPC patients may have symptoms such as bleeding in the nose, tinnitus and nasal congestion, etc. However, due to the lack of specificity of early symptoms, approximately 70% of patients at the first diagnosis have entered locally advanced stage [2]. Moreover, the rates of local recurrence and distant metastasis are high, and the survival time is much lower than that of early NPC patients [3].

Long noncoding RNAs (lncRNAs) play a crucial part in the development and progression of cancer [4]. The evolutionary conservatism, diversity and complexity of lncRNAs indicate that they have a vital role in regulating cell growth. Thus, it might be a gold mine to study lncRNAs for basic scientific research, biomarker and drug discovery, and potential therapies. Besides, lncRNAs regulate the development and progression of NPC [5,6]. Therefore, it is necessary to identify specific NPC-related lncRNA molecules and determine strategies for their clinical application.

It is worth noting that, as a tumor suppressor or oncogenic molecule, LncRNA RGMB antisense RNA 1 (RGMB-AS1) refers to the regulation of malignant tumors during the progression of many cancers [7]. For example, lncRNA RGMB-AS1, as an oncogenic molecule, is highly expressed in human non-small cell lung cancer [8]. LncRNA RGMB-AS1 also participates in the development of papillary thyroid carcinoma [9]. Moreover, lncRNA RGMB-AS1 plays an anticancer role in hepatocellular carcinoma [10]. However, the role of lncRNA RGMB-AS1 in NPC remains unknown. Importantly, we proved the binding mode of lncRNA RGMB-AS1 and Forkhead box A1 (FOXA1) through RIP experiment in our previous study, so we speculated that FOXA1 might be involved in the regulatory effect of lncRNA RGMB-AS1 on NPC development. Researches show that FOXA1 expression is decreased in NPC samples, and the up-regulation of FOXA1 in NPC cells inhibits the tumorigenicity in *vivo* [11,12]. The overexpression of FOXA1 is associated with a good prognosis in NPC patients. FOXA1 plays an important role in epithelial mesenchymal transformation (EMT) through key factors such as E-cadherin, and a positive correlation between E-cadherin and FOXA1 expression was observed in these studies [13]. In summary, although these data suggest that FOXA1 regulates NPC development, further study are required to elucidate the mechanism of its effect on NPC development. Since lncRNA RGMB-AS1 could directly bind to FOXA1 in NPC, this study focuses on the new mechanism of lncRNA RGMB-AS1 on NPC biological function and EMT by regulating FOXA1.

Here, we aimed to evaluate the expression of lncRNA RGMB-AS1 in NPC and explore the new mechanisms of lncRNA RGMB-AS1/FOXA1 axis on biological function and EMT in NPC, to determine whether lncRNA RGMB-AS1 can be used as a gene therapy target for NPC in *vitro* and in *vivo*. As a result, we particularly emphasize the new mechanism of lncRNA RGMB-AS1 regulating FOXA1 on biological function and EMT of NPC. We found low expression of lncRNA RGMB-AS1 in NPC tissues and cells. The knockdown of lncRNA RGMB-AS1 promoted tumor proliferation and EMT, while the overexpression of lncRNA RGMB-AS1 had the opposite effect. Further studies showed that lncRNA RGMB-AS1 inhibited tumor proliferation and EMT by directly binding to FOXA1. Therefore, we identified a new potential therapeutic target for NPC treatment.

## 2 Materials and methods

### 2.1 Clinical samples

Thirty nasopharyngeal carcinoma patients with complete clinical data who received radical nasopharyngeal carcinoma resection were collected from the Department of General Surgery of the Affiliated Hospital of Guizhou Medical University between February 2019 and February 2020. The nasopharyngeal cancer tissue of patients was set as the experimental group, and the corresponding paracancerous tissue of nasopharyngeal cancer was set as the normal control group. The extracted tissue was immediately frozen in liquid nitrogen at −80°C. The clinical samples was approved by the local Institution Review Board of the Affiliated Hospital of Guizhou Medical University (approval No. ChiCTR-IPR-14005772). Patients and their families participated in the study voluntarily and signed the informed consent.

### 2.2 Cell cultures and cell transfection

Normal nasopharyngeal epithelial cell line (NP69) and 4 nasopharyngeal carcinoma cell lines (5–8 F, C666-1, CNE-2, and HNE2) were purchased from Wuhan Prosera Life Science and Technology Co., Ltd., while 1 nasopharyngeal carcinoma cell line (6–10B) was purchased from Beijing Beina Institute of Biotechnology. All cells were cultured in Dulbecco modified Eagle medium (DMEM) containing 10% fetal bovine serun (FBS), 100 U/mL penicillin, 100 μg/mL streptomycin. For selection of efficient lncRNA RGMB-AS1 siRNA, the Opti-MEM (Invitrogen, Grand Island, NY, USA) was used. The following siRNAs were transfected on the basis of the Lipofectamine 3000 protocol (Lipo3000, Invitrogen): Negative control of lncRNA RGMB-AS1: 5′-GCUUUACCAGCUCGCUAAUUU-3′; lncRNA RGMB-AS1-1**#**: 5′-GCUUGGCGUGAGCAAUGCAUU-3′; lncRNA RGMB-AS1-2#: 5′-GCAGCUCGUGGUUUGUACUUU-3′; Negative control of FOXA1: 5′-UUCUCCGAACGUGUCACGUTT-3′; FOXA1 siRNA-1**#**: 5′-GAGAGAAAAAAUCAACAGCTT-3′; FOXA1 siRNA-2**#**: 5′-CCGGUCAGCAACAUGAACUTT-3′. The cells were gathered after 72 h for mRNA quantification. In *vitro* studies used siRNA with the best inhibitory effect, and clone formation assay and nude mouse xenograft studies used the lncRNA RGMB-AS1 shRNA or FOXA1 shRNA lentivirus [14].

### 2.3 Constructing lentivirus-mediated lncRNA RGMB-AS1/FOXA1 shRNA and lncRNA RGMB-AS1 pcDNA

The pLKO.1-shRNA lncRNA RGMB-AS1/FOXA1 and their control plasmids, as well as packaging plasmids including psPAX2 and PMD2G, were designed and cloned. The untransfected cells were eliminated with 2 µg/mL puromycin (Sigma-Aldrich, St-Louis, Missouri, USA) for 3 weeks to obtain stable cell lines [15]. These lentivirus-based constructs were co-transfected to the cultured CNE-2 and 6–10B cells. 72 h later, the replication-deficient lentivirus particles were packaged and collected for in *vivo* experiment.

### 2.4 RT-qPCR

RNA was extracted from cells or tissues using Trizol reagent (Invitrogen, Carlsbad, CA, USA). Briefly, we synthesized cDNA through the Maxima first strand cDNA synthesis kit (Sangon Biotech, Shanghai, China). The ABI 7500 RT-PCR system (Applied Biosystems, Foster City, CA, USA) and the SYBR Premix Ex Taq kit (Biosteel Biotechnology, Shanghai, China) were used for RT-qPCR [16,17]. LncRNA RGMB-AS1 was normalized to GAPDH, while FOXA1, Vimentin and E-cadherin were normalized to β-actin. The primer sequences of RT-qPCR were shown in Table 1.

**Table 1.** The primers used in this study for RT-PCR

gene	sequence (5′→3′)
Lnc RGMB-AS1	F-AGTGGGCAAACTTCAACGTTC
	R-GAGCTGCCATTGAATTAATCCG
GAPDH	F-GAGTCCACTGGCGTCTTC
	R-GATGATCTTGAGGCTGTTGTC
FOXA1	F-AGGGCTGGATGGTTGTATTG
	R-GCCTGAGTTCATGTTGCTGA
Vimentin	F- GCCCTAGACGAACTGGGTC
	R- GGCTGCAACTGCCTAATGAG
E-cadherin	F- TGATTCTGCTGCTCTTGCTGTT
	R- CCTCTTCTCCGCCTCCTTCTT
β-actin	F-CAAGGCCAACCGCGAGAA
	R-CCCTCGTAGATGGGCACAGT

### 2.5 Nuclear/plasmic separation of cells

RNA Subcellular Isolation was used to isolate RNA from the cytoplasm or nucleus of nasopharyngeal cancer cells through the RNA Subcellular Isolation Kit (Active Motif, Carlsbad, CA, USA) on the basis of the corresponding instructions. In short, we collected CNE-2 and 6–10B cells and lysed them on ice for 5 minutes. The cells were then centrifuged at 12000 g for 3 minutes. Finally, the supernatant was gathered and the cytoplasmic RNA was measured, while nuclear RNA was extracted using nuclear precipitation [18].

### 2.6 CCK-8

Cell viability was monitored using the Cell Counting Kit-8 assay [19] (LaiFuSai Technology, Nanjing, Jiangsu, China). After transfection, CNE-2 and 6–10B cells were grown in 96-well plates. According to the corresponding instructions, we measure cell viability every 24 hours. Finally, the absorbance was read at 450 nm using Powerwave 340 (BioTek Instruments, Winooski, VT, USA).

### 2.7 Colony experiments

CNE-2 cells and 610-B cells from RPMI-1640 containing 10% FBS were spread into the 6-well plate and cultured in a constant temperature incubator at 5% CO_2_ and 37°C for 2 weeks. The medium needs to be replaced every three days. After 2 weeks, the colonies were fixed with methanol for 30 min and stained with 0.1% crystal violet (Sigma-Aldrich, USA) for 15 min. Colony counts were calculated using a light microscope (Olympus, Tokyo, Japan) [20].

### 2.8 Transwell migration experiment

The migration ability of CNE-2 cells and 610-B cells was determined by Transwell [19]. About 5 × 10^4^ cells were inoculated into the upper chambers of transwell inserts in RPMI-1640 medium. The lower chamber was then covered with the medium containing 8% FBS. After incubation for 24 h, the cells were fixed with crystal violet. Subsequently, the cells were counted under a microscope.

### 2.9 Western blot

The logarithmic growth cells were collected, lysed with RIPA, and the total protein was extracted. Adjust the protein concentration to 4 μg/μL with BCA kit. The total proteins were then isolated by SDS-PAGE and transferred to PVDF membranes (EMD Millipore, Burlington, MA, USA). Finally, protein antibodies were used overnight at 4°C. Primary antibodies: E-cadherin (1:1000), Vimentin (1:1000), FOXA1 (1:1000) and β-actin (1:100,000) were purchased from Abcam (Cambridge, UK). After incubation with sheep anti-rabbit antibody for 1 h at room temperature, the membrane was treated with ECL luminescent solution. Protein expression was represented by gray value [21].

### 2.10 RIP

Based on the experimental protocol, RNA immunoprecipitation was performed using the Thermo Fisher RIP kit (Thermo Fisher Scientific, Waltham, Massachusetts, USA). FOXA1 antibodies were purchased from Abcam (Cambridge, UK). We treated normal rabbit IgG (Abcam) as the negative control of the RIP procedure [22]. After the target RNA was extracted and purified, the purified RNA was examined by RT-qPCR and Western blot analysis to verify the presence of its binding target.

### 2.11 Animal experiments

Eighteen BALB/c nude mice aged 4–6 weeks and weighing 20 ± 2 g were purchased from Animal Center of Guizhou Medical University. They were kept in a specefic Pathogen free (SPF) room in the school’s animal house. The experiment was approved by the school experimental Animal Ethics Committee. These nude mice were randomly divided into 3 groups: pcDNA-NC group, pcDNA-RGMB-AS1-1# group and pcDNA-RGMB-AS1-2# group. In the subcutaneous carcinogenic experiment, the right axillas of nude mice in each NC group were subcutaneously injected with stably transfected pcDNA-NC CNE-2 cells. The pcDNA-RGMB-AS1-1# or pcDNA-RGMB-AS1-2# group were respectively injected into the constructed pcDNA-RGMB-AS1-1# or pcDNA-RGMB-AS1-2# CNE-2 cells. Each group were injected with about 4 × 10^6^ cells. 4 weeks after the successful establishment of the model, the nude mice were anesthetized by intraperitoneal injection of pentobarbital sodium (50 mg/kg). The animals were removed from the cervical vertebra and sacrificed. Tumor tissues were taken out for weighing and volume measurement [23]. Later, some tumors were placed in liquid nitrogen quick-freezing and stored in a refrigerator at −80°C for future use. All animal experiment procedures had been approved by the Animal Care & Welfare Committee of GuiZhou Medical University (approval No. 2101395).

### 2.12 Immunohistochemical (IHC) staining

IHC was conducted using the two-step EnVision method [24]. The determination was carried out in accordance with the manufacturer’s instructions. Anti-Ki-67 (Abcam; ab15580; 1:200), anti-Vimentin (Abcam; ab92547; 1:1000) and anti-E-Cadherin (Abcam; ab92547; 1:1000) antibody were used to stain the tissues, and then stained in the transplanted tumor tissues transfected with pcDNA-NC or pcDNA-RGMB-AS1. Finally, the optical density (OD) of the sections was measured.

### 2.13 Statistical analysis

Data results were obtained using SPSS 21.0 software (SPSS Inc., Chicago, IL, USA). All data were expressed as mean ± standard deviation (mean ± SD). The comparison between the two groups was performed by Student’s T test. One-way ANOVA test was used for comparison between multiple groups, LSD test was used for homogeneity of variance, and Tamhane’s T2 test was used for heterogeneity of variance. *P* < 0.05 was considered as significant difference between groups.

## 3 Results

### 3.1 LncRNA RGMB-AS1 and FOXA1 mRNA levels were down-regulated in NPC tissues and cells.

**Figure 1.**
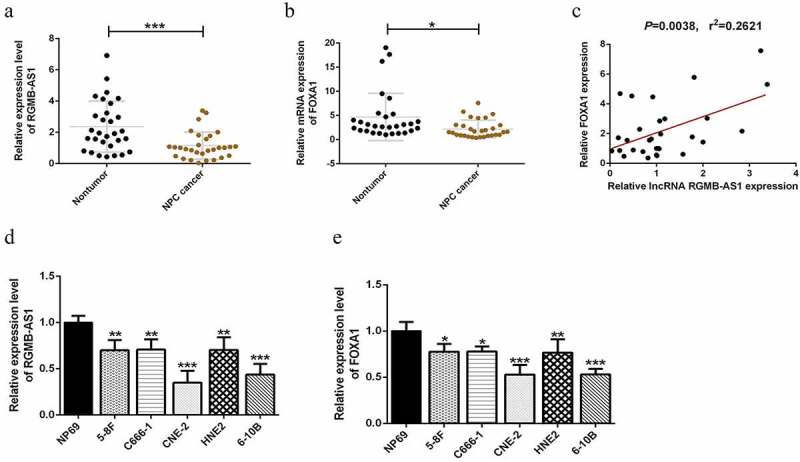
LncRNA RGMB-AS1 and FOXA1 mRNA levels were down-regulated in NPC tissues and cells. (a) Expression level of lncRNA RGMB-AS1 in nasopharyngeal carcinoma tissues and corresponding paracancerous tissues of 30 patients with nasopharyngeal carcinoma (n = 30, ***P < 0.001); (b) FOXA1 expression levels in nasopharyngeal carcinoma tissues and corresponding paracancerous tissues of 30 patients with nasopharyngeal carcinoma (n = 30, *P < 0.05); (c) RT-qPCR assay for the correlation between lncRNA RGMB-AS1 level and FOXA1 level; (d-e) The expression levels of lncRNA RGMB-AS1 and FOXA1 in human immortalized normal nasopharyngeal epithelial cell line (NP69) and 5 nasopharyngeal carcinoma cell lines (5–8 F, C666-1, CNE-2, HNE2 and 6–10B) (n = 3, *P < 0.05, **P < 0.01 vs NP69 group). Data were expressed as mean ± SD.

In order to further explore the roles of lncRNA RGMB-AS1 and FOXA1 in NPC development for elucidating the potential mechanism of lncRNA RGMB-AS1 on NPC, here, we firstly focused on the expressions of lncRNA RGMB-AS1, FOXA1 and EMT-related markers in nasopharyngeal carcinoma and their related clinical significance. We analyzed the mRNA expressions of lncRNA RGMB-AS1 and FOXA1 in nasopharyngeal carcinoma tissues and adjacent tissues of 30 patients with nasopharyngeal carcinoma who underwent radical NPC resection. The RT-qPCR results showed that the expression levels of lncRNA RGMB-AS1 and FOXA1 in nasopharyngeal carcinoma tissues were significantly lower than those in their paired paracancerous tissues (Figures 1A and B). Besides, lncRNA RGMB-AS1 level was found to be positively correlated with FOXA1 level (Figure 1C). We treated NP69 as the normal control group and 5 nasopharyngeal carcinoma cell lines as the experimental group. By contrast, the levels of lncRNA RGMB-AS1 and FOXA1 in nasopharyngeal carcinoma cells were significantly lower than those in NP69 cells (Figures 1D and E). As shown in Table 2, a distinct relationship was found between low lncRNA RGMB-AS1 expression and invasive potential of NPC, and lncRNA RGMB-AS1 expression in tumors was significantly correlated with the presence of Vimentin mutation and E-cadherin mutation. The data highlighted the role of lncRNA RGMB-AS1 as a potential biomarkers in NPC.

**Table 2.** Correlation between lncRNA RGMB-AS1 expression and clinicopathological factors

Factors	Cases	lncRNA RGMB-AS1(%)		*χ2*	*P*-Value
		Low	High		
Age	≤60 (22)	12 (80)	10 (67)	0.682	0.682
	≥60 (8)	3 (20)	5 (33)		
Gender	Male (21)	14 (93)	7 (47)	7.778	0.014*
	Female (9)	1 (7)	8 (53)		
Clinical stage	I–II (8)	1 (7)	7 (47)	6.136	0.035*
	III–IV (22)	14 (93)	8 (53)		
Pathological classification	Squamous cell carcinoma (25)	11 (73)	14 (93)	2.160	0.330
	Undifferentiated non-keratinocarcinoma (5)	4 (27)	1 (7)		
Degree of histological differentiation	High and middle (8)	0 (0)	8 (53)	10.909	0.002**
	Low (22)	15 (100)	7 (47)		
Lymph node metastasis	Present (18)	14 (93)	4 (27)	13.889	<0.001***
	Absent (12)	1 (7)	11 (73)		
Vimentin mutation	No (11)	1 (7)	8 (53)	7.778	0.014*
	Yes (19)	14 (93)	7 (47)		
E-cadherin mutation	No (9)	12 (87.5)	4 (50)	8.571	0.009**
	Yes (21)	3 (12.5)	11 (50)		

*P < 0.05, **P < 0.01, ***P < 0.001.

### 3.2 LncRNA RGMB-AS1 is closely related to the expression levels of E-cadherin and Vimentin in NPC tissues

**Figure 2.**
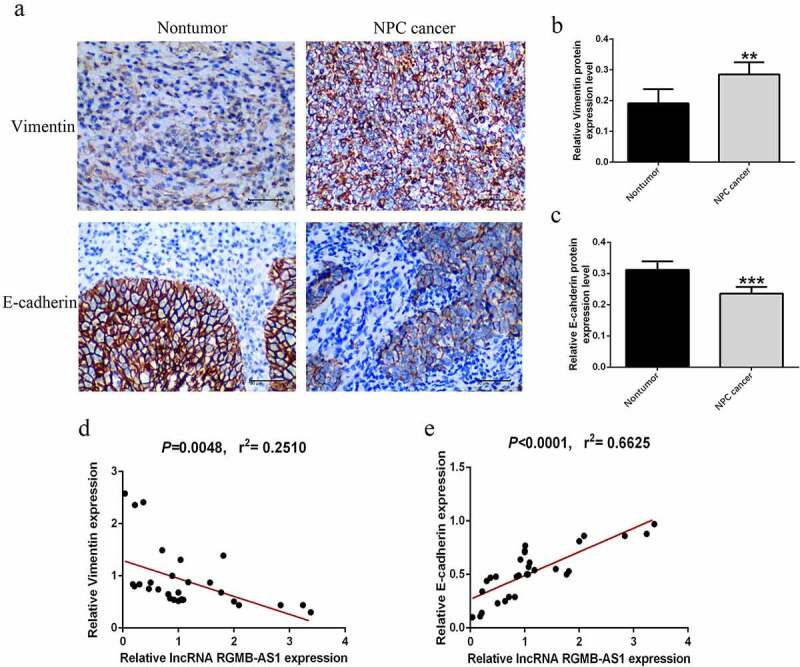
LncRNA RGMB-AS1 was closely related to the expression levels of E-cadherin and Vimentin in NPC tissues. (a-c) Expression of EMT-related factors (E-cadherin and Vimentin) in NPC and para-cancer tissues (n = 6, **P < 0.01, ***P < 0.001). (d) Correlation between lncRNA RGMB-AS1 expression level and Vimentin expression level in NPC tissues. (e) Correlation between lncRNA RGMB-AS1 expression level and E-cadherin expression level in NPC tissues.

In order to preliminarily explore whether the EMT process occurred in these NPC tissues, the expression levels of E-cadherin and Vimentin in NPC tissues and their paired paracancer tissues were detected by immunohistochemistry. The results showed that the expression of Vimentin in NPC tissues was significantly increased, while E-cadherin was significantly decreased (Figures 2A-C). Next, we explored the expression levels of E-cadherin and Vimentin in NPC tissues by RT-qPCR, and analyzed their correlation with the expression of lncRNA RGMB-AS1 respectively. We found that E-cadherin expression in NPC was significantly positively correlated with lncRNA RGMB-AS1 expression, while Vimentin expression was significantly negatively correlated with lncRNA RGMB-AS1 expression (Figures 2D and E).

### 3.3 Effects of overexpression or knockdown of lncRNA RGMB-AS1 on cell proliferation and migration

**Figure 3.**
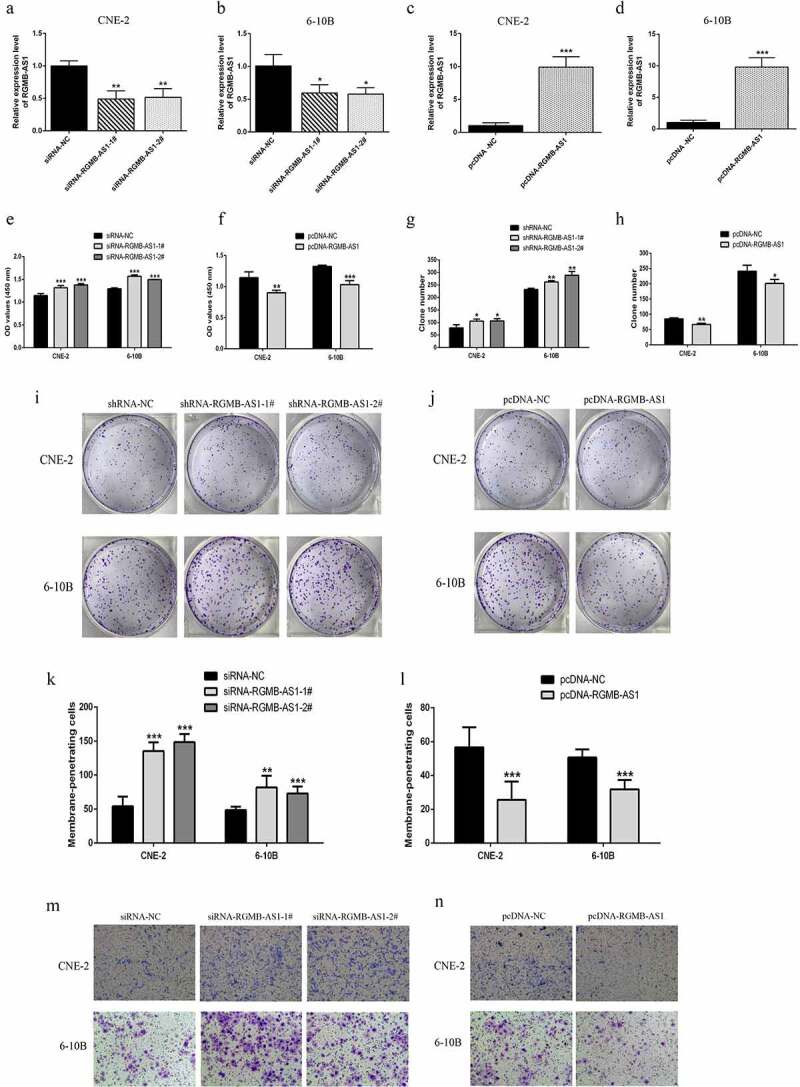
Effects of overexpression or knockdown of lncRNA RGMB-AS1 on cell proliferation and migration. (a – d) Expression level of lncRNA RGMB-AS1 in CNE-2 cells (a and c) and 6–10B cells (b and d); (e-f) CCK-8 assay was used to detect the effect of lncRNA RGMB-AS1 on cell proliferation. (g-j) Clone formation assay was used to detect the effect of lncRNA RGMB-AS1 cell proliferation. (k-n) Transwell chamber assay was used to detect the effect of lncRNA RGMB-AS1 on cell migration. (mean ± SD, n = 3; *p < 0.05, **p < 0.01, ***p < 0.001 vs the corresponding negative control group).

To further investigate whether the up-regulation of lncRNA RGMB-AS1 expression was related to the inhibition of cell biological behavior in NPC, while the silencing of lncRNA RGMB-AS1 expression had the opposite effect, we silenced and overexpressed lncRNA RGMB-AS1, and preliminarily confirmed the possible mechanism of lncRNA RGMB-AS1 on NPC cell biological behavior. Firstly, the overexpression and silencing effect of lncRNA RGMB-AS1 in CNE-2 cells and 6–10B cells were detected by RT-qPCR. The results showed that the expression of lncRNA RGMB-AS1 was significantly decreased after transfection with siRNA-RGMB-AS1-1#/2#, while the expression of lncRNA RGMB-AS1 was significantly increased after transfection with pcDNA-RGMB-AS1 both in CNE-2 and 6–10B cells (Figures 3A-D). Then CCK-8 was used to detect the effect of lncRNA RGMB-AS1 on cell proliferation. The results showed that silencing lncRNA RGMB-AS1 expression significantly increased cell proliferation, while overexpressing lncRNA RGMB-AS1 significantly inhibited cell proliferation (Figures 3E and F). In addition, lentiviral vector-mediated shRNA carrying stable silencing lncRNA RGMB-AS1 gene and vector-mediated pcDNA carrying stable overexpressing lncRNA RGMB-AS1 cell lines were established. Subsequently, clone formation experiment was used to assess the influence of lncRNA RGMB-AS1 on cell clone forming ability. The results showed that lowly expressed lncRNA RGMB-AS1 in CNE-2 and 6–10B cells promoted cell clone formation, while highly expressed lncRNA RGMB-AS1 inhibited cell clone formation (Figures 3G and J).

We evaluated the effect of lncRNA RGMB-AS1 on cell migration. Transwell invasion assay showed that the migration ability of cells in the siRNA-RGMB-AS1-1#/2# group was significantly higher than that in the siRNA-NC group. However, the migration ability of cells in the pcDNA-RGMB-AS1 group was significantly lower than that in the pcDNA-NC group (Figures 3K-N).

### 3.4 Effects of overexpression or knockdown of lncRNA RGMB-AS1 on cell EMT process

**Figure 4.**
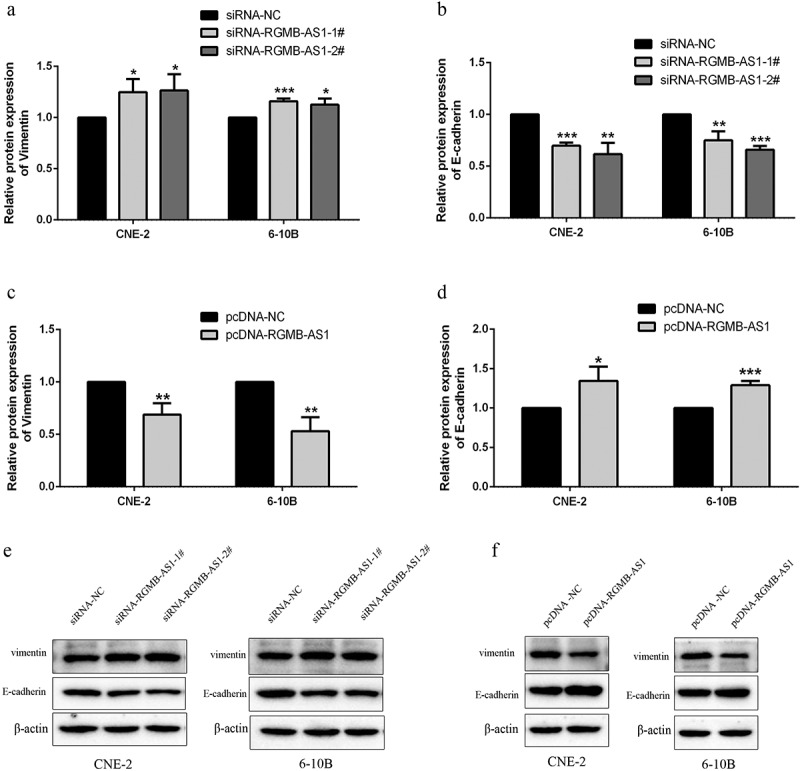
Effects of overexpression or knockdown of lncRNA RGMB-AS1 on cell EMT process. (a-b) The effect of lncRNA RGMB-AS1 silencing on Vimentin(a) and E-cadherin(b) expression; (c-d) Effect of lncRNA RGMB-AS1 overexpression on Vimentin(c) and E-cadherin(d) expression; (e-f) Images of lncRNA RGMB-AS1 silencing (e) or overexpression (f) regulated Vimentin and E-cadherin expression levels. (mean ± SD, n = 3; *p < 0.05, **p < 0.01, ***p < 0.001 vs the corresponding negative control group).

For investigating whether the change of lncRNA RGMB-AS1 level affected the EMT of NPC, we performed Western blot detection. The results showed that, in both of the lowly expressed lncRNA RGMB-AS1 groups, the Vimentin expression levels were significantly increased (Figures 4A and E), while the expression level of E-cadherin was significantly decreased (Figures 4B and E). Furthermore, in the highly expressed lncRNA RGMB-AS1 group, the expression level of Vimentin was significantly decreased (Figures 4C and F), and the expression level of E-cadherin was significantly increased (Figures 4D and F).

### 3.5 Effects of the acquisition or loss of lncRNA RGMB-AS1 function on FOXA1 expression

**Figure 5.**
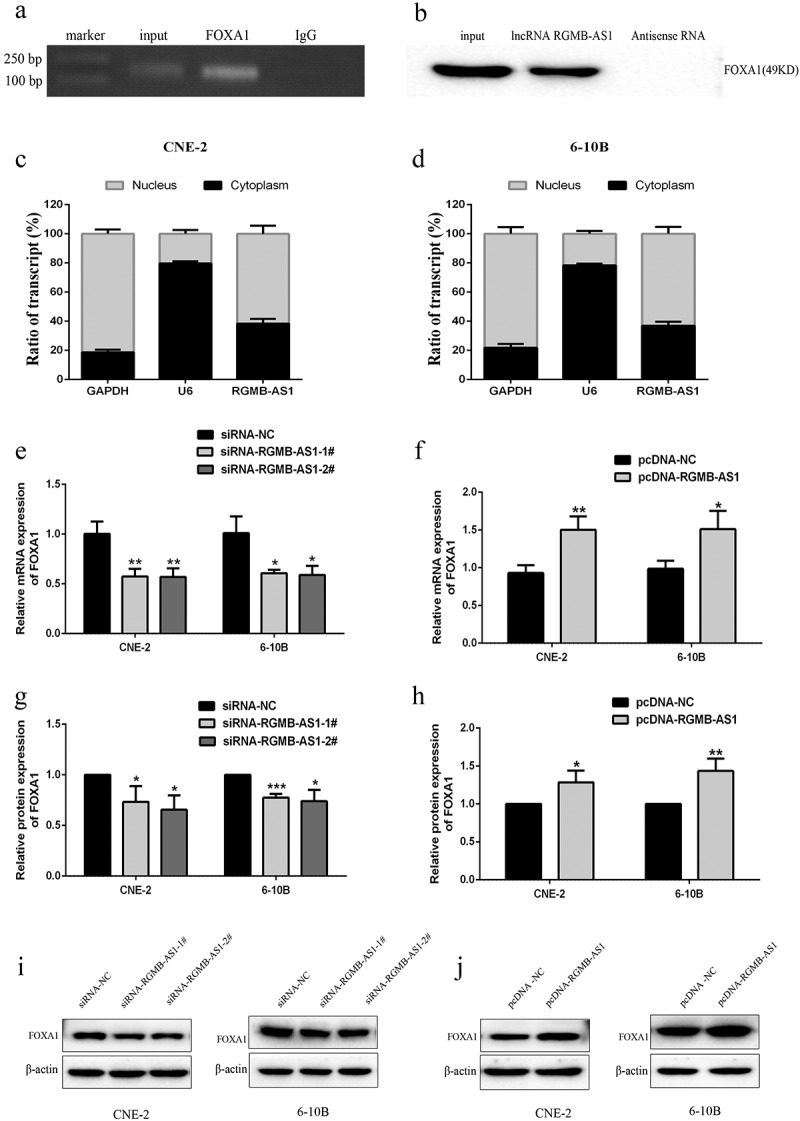
Effects of the acquisition or loss of lncRNA RGMB-AS1 function on FOXA1 expression. (a-b) Determination of the ability of FOXA1 to interact with lncRNA RGMB-AS1 via RIP assay. (c-d) Distribution of lncRNA RGMB-AS1 in the nucleus and cytoplasm of CNE-2 cells (c) and 6–10B cells (d); (e-f) Effects of silencing (e) or overexpression (f) of lncRNA RGMB-AS1 on FOXA1 mRNA expression; G-H. Effects of silencing (g) or overexpression (h) of lncRNA RGMB-AS1 on FOXA1 protein expression; (i-j) Images of silencing (i) or overexpression (j) of lncRNA RGMB-AS1 regulating FOXA1 expression level. (mean ± SD, n = 3; *p < 0.05, **p < 0.01, ***p < 0.001 vs the corresponding negative control group).

In order to further understand the epigenetic regulation potential mechanisms of lncRNA RGMB-AS1 on FOXA1 expression, RIP detection was used to confirm that lncRNA RGMB-AS1 could directly bind FOXA1 in NPC cells (Figures 5A and B). Subsequent RT-qPCR was performed to detect the distribution of lncRNA RGMB-AS1 in the nucleus and cytoplasm. The results showed that lncRNA RGMB-AS1 existed in both cytoplasm and nucleus, but was mainly enriched in nucleus, accounting for about 2/3 of the total amount (Figures 5C and D).

Next, we used RT-qPCR and Western blot to detect the effect of silencing or overexpressing lncRNA RGMB-AS1 on the expression level of FOXA1. The results showed that mRNA (Figure 5E) and protein (Figures 5G and I) expressions of FOXA1 were both significantly lower in cells transfected with the lowly expressed lncRNA RGMB-AS1 than those of cells transfected with siRNA-NC. Besides, the levels of FOXA1 mRNA (figure 5F) and protein (Figures 5H and J) were significantly higher in cells transfected with the highly expressed lncRNA RGMB-AS1 than those of cells transfected with pcDNA-NC.

### 3.6 FOXA1 knockdown reversed the inhibition of lncRNA RGMB-AS1 on cell proliferation, migration and EMT process

**Figure 6.**
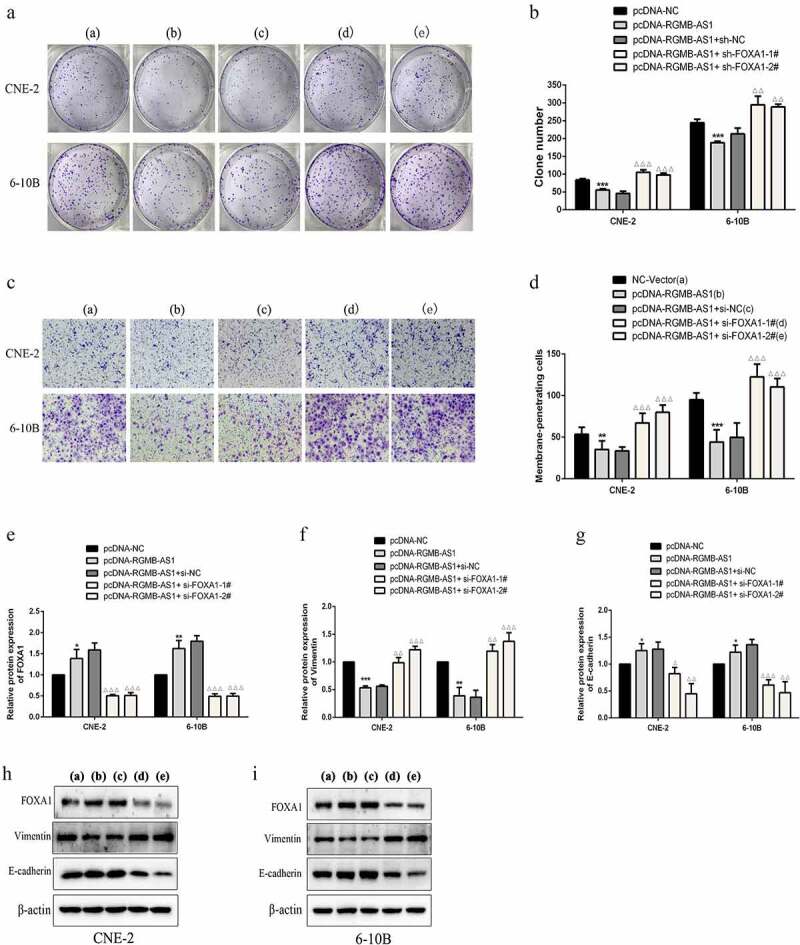
FOXA1 knockdown reversed the inhibition of lncRNA RGMB-AS1 on cell proliferation, migration and EMT process. (a-b) The effect of lncRNA RGMB-AS1 on the clone formation ability of NPC cells by regulating FOXA1. (c-d) The effect of lncRNA RGMB-AS1 on the migration ability of NPC cells by regulating FOXA1. (e-g) The effect of lncRNA RGMB-AS1 on the expression levels of FOXA1 (e) Vimentin (f) and E-cadherin (g) by regulating FOXA1. (h-i) Image of lncRNA RGMB-AS1 regulating FOXA1 expression levels on FOXA1, Vimentin and E-cadherin proteins. (mean ± SD, n = 3; *P < 0.05, **P < 0.01, ***p < 0.001 vs the corresponding negative control group; ^Δ^*P*<0.05, ^ΔΔ^*P*<0.01, ^ΔΔΔ^*P*<0.001 vs pcDNA-RGMB-AS1+ si-NC group).

Subsequently, the effects of lncRNA RGMB-AS1 and FOXA1 on cell proliferation and migration were assessed in *vitro*. Results indicated that after overexpressing lncRNA RGMB-AS1 in both CNE-2 and 6–10B cells, colony formation (Figures 6A and B) and migration (Figures 6C and D) were significantly reduced, while FOXA1 knockdown reversed its reduction effect. These results suggested that lncRNA RGMB-AS1 inhibited the proliferation and migration of NPC cells by promoting FOXA1.

We determined whether the lncRNA RGMB-AS1/FOXA1 regulatory axis regulated EMT in NPC cells. Firstly, Western blotting were employed to examine FOXA1 expression and indicated that overexpression of lncRNA RGMB-AS1 promoted FOXA1 expression, whereas FOXA1 siRNA showed a partial reversal effect on the promoting effect of overexpression of lncRNA RGMB-AS1 (Figures 6E, H and I). Meanwhile, the expressions of EMT markers were determined. The results showed that the overexpression of lncRNA RGMB-AS1 decreased expression of Vimentin and increased E-cadherin expression in NPC cells, while the inhibition of FOXA1 partially offset this effect. (Figures 6F-I). These results indicated that the lncRNA RGMB-AS1/FOXA1 regulatory axis inhibited the EMT of NPC cells.

### 3.7 The effect of lncRNA RGMB-AS1 on xenograft growth in NPC nude mice

**Figure 7.**
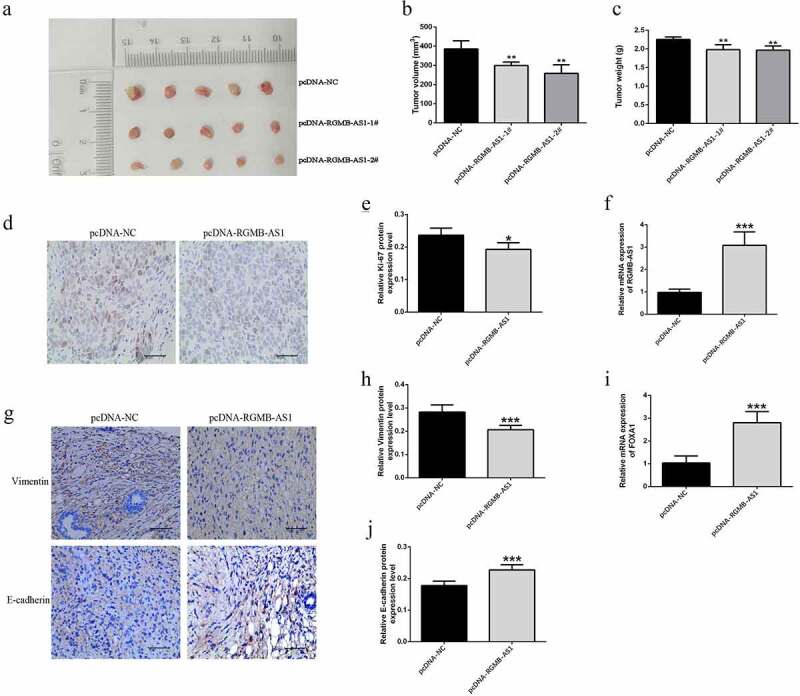
The effect of lncRNA RGMB-AS1 on FOXA1 xenograft growth in NPC nude mice. (a) Morphology of NPC transplanted tumor in nude mice; (b) The size of NPC transplanted tumor in nude mice; (c) Expression level of lncRNA RGMB-AS1 in NPC xenograft of nude mice; (d) The expression level of FOXA1 in NPC xenograft of nude mice; (e) The expression level of Ki-67 protein in NPC transplanted tumor tissue; (f) Immunohistochemical image of Ki-67 protein expression. (mean ± SD, n = 6; *P < 0.05, **P < 0.01).

In order to further investigate the effect of lncRNA RGMB-AS1 overexpression on NPC growth in *vivo*, the CNE-2 cell line with stable lncRNA RGMB-AS1 overexpression was constructed using pcDNA-RGMB-AS1, and then subcutaneously injected into the right axilla of nude mice to establish the CNE-2 xenograft tumor model in nude mice. The results showed that compared to the control treatment, the tumor volume and the tumor weight in the pcDNA-RGMB-AS1-1# and 2# group were significantly reduced. It suggested that the growth rate of nasopharyngeal carcinoma CNE-2 cell lines after overexpression of lncRNA RGMB-AS1 was lower than that of the negative control group in *vivo* (Figures 7A-C). In addition, the expressions of Ki-67 were decreased in nude mice transplanted tumor after overexpression of lncRNA RGMB-AS1 (Figures 7D and E). These results suggested that up-regulation of lncRNA RGMB-AS1 led to reduced NPC growth.

Importantly, the expressions of lncRNA RGMB-AS1 and FOXA1 were all significantly increased in nasopharyngeal carcinoma xenograft mice with overexpression of lncRNA RGMB-AS1 (Figures 7F and 7I). Vimentin expression level in pcDNA-RGMB-AS1 group was significantly lower than that in pcDNA-NC group (Figures 7G and H), while E-cadherin expression level in pcDNA-RGMB-AS1 group was significantly higher than that in pcDNA-NC group (Figures 7G and J). These results were consistent with our results in *vitro.*

## 4 Discussion

In recent years, radiotherapy technology has been further developed, but the 5-year survival rate of NPC patients has not improved very well. Therefore, how to improve the therapeutic effect of NPC is an urgent problem to be solved at present, and efforts should be made in many aspects, such as early diagnosis and finding new therapeutic targets. A great deal of studies have shown that there are some changes of lncRNA expressions in NPC, and some of the identified lncRNAs have important implications for regulating the occurrence of NPC [25]. As described in a prior study, lncRNA RGMB-AS1 is involved in the regulation of malignant tumors during the progression of many cancers [7]. However, the role of lncRNA RGMB-AS1 in NPC remains largely unknown. Therefore, this study was mainly to explore the potential mechanism of lncRNA RGMB-AS1 affecting the biological behavior of NPC cells. The results of this study showed that lncRNA RGMB-AS1 acted as an up-regulated FOXA1 sponge, inhibiting malignant biological behavior and EMT process of NPC cells. Our findings provided a new theoretical basis for clinical application.

NPC has the biological characteristics of high invasiveness and high metastasis [26]. Therefore, the control of metastasis is a key factor to improve the prognosis and prolong the survival time of tumor patients. Studies had shown that lncRNAs regulated the occurrence and development of different types of cancer, including NPC [27,28]. It had been found that lncRNA RGMB-AS1 was also involved in the proliferation, migration and other malignant biological behaviors of various tumors [10,29]. Here, we emphasized the role of lncRNA RGMB-AS1 in NPC. We confirmed that the expression of lncRNA RGBM-AS1 in NPC tissues was significantly lower than that in paracancerous tissues, and this result was also confirmed in 5 cell lines. Then, we provided evidence that overexpression of lncRNA RGMB-AS1 significantly inhibited the proliferation and migration of NPC cells, while knockdown of lncRNA RGMB-AS1 showed the opposite effect. In recent years, some lncRNAs had been proved to affect the occurrence and development of cancer through regulatory mechanism in NPC. For example, lncRNA MSC-AS1 promoted the proliferation of hepatoma carcinoma cells by maintaining PFKFB3 expression [30]. lncRNA RBM24 inhibited NPC development by targeting MALAT1 [31]. These studies suggested that it was of great significance to identify the regulatory mechanisms of specific lncRNAs in NPC for exploring new therapeutic targets. Here, we investigated the subcellular localization of lncRNA RGMB-AS1 in NPC. As is known to all, specific roles of lncRNAs are different when they are in different subcellular localization. Therefore, the function of lncRNAs are related to their unique subcellular localization. For example, lncRNAs in the cytoplasm were mainly involved in gene regulation at the post-transcriptional and translational levels, including interacting with the cytoplasm proteins [32], binding with target mRNA to inhibit their translation [33], and acting as endogenous competing RNAs (ceRNAs) that interact with microRNAs [34]. LncRNAs located in the nucleus were mainly involved in gene regulation at epigenetic and transcriptional levels, including protein regulation in the cell nucleus [35]. We found that lncRNA RGMB-AS1 existed in both cytoplasm and nucleus through plasmic nucleus separation experiments, suggesting that lncRNA RGMB-AS1 may be involved in the malignant biological behavior of NPC through two different pathways: cytoplasm and nucleus. LncRNA RGMB-AS1 mainly distributed in the nucleus, which further suggested that lncRNA RGMB-AS1 inhibited the process of NPC mainly through protein interaction in the nucleus. Subsequently, we confirmed the binding relationship between lncRNA RGMB-AS1 and FOXA1, suggesting a regulatory relationship between them. In this study, further experiments confirmed that lncRNA RGMB-AS1 inhibited the proliferation, migration and EMT of NPC cells by positively regulating FOXA1 gene expression. The same results were confirmed by our in *vivo* tumor transplant experiment.

In-depth study of lncRNA on EMT process in cancer is of great significance for investigating cell biological behavior [36,37]. EMT is a process in which epithelial cells are transformed into mesenchymal cells by characteristic loss, with decreased expression of epithelial markers (such as E-cadherin) and increased expression of mesenchymal markers (such as Vimentin) [38]. EMT gave the ability of cancer cells to migrate and invade [39], and a large number of lncRNAs were involved in the progress of EMT in NPC [6,40]. E-cadherin acted as a negative regulator of EMT, while Vimentin acted as its positive regulator. Here, FOXA1 knockdown reversed the increased expression of the EMT marker E-cadherin and decreased expression of Vimentin in NPC cells induced by overexpression of lncRNA RGMB-AS1. FOXA1 had also been reported to play a role in a variety of cancers [41,42]. Previous studies have reported that FOXA1 was highly expressed in the normal nasopharyngeal epithelium, but was reduced in NPC samples [11,12]. Restoration of FOXA1 in NPC cells inhibited cell proliferation and invasion in *vitro* and tumorigenicity in *vivo* [43,44]. In addition, FOXA1 played an important role in the EMT process of NPC through key factors such as E-cadherin. A positive correlation between E-cadherin and FOXA1 expression was observed in these studies [13]. Several other studies showed that FOXA1 promoted EMT mainly by regulating E-cadherin expression [45,46]. Similar results were fully demonstrated in our study.

These findings suggest that FOXA1 was involved in the inhibition of the proliferation, migration and EMT of lncRNA RGMB-AS1 in NPC cells. Further experiments on xenografts in nude mice showed that lncRNA RGMB-AS1 inhibited tumor growth, which was consistent with in *vitro* results. Overexpression of lncRNA RGMB-AS1 restricted the growth of CNE-2 xenografts in nude mice and promoted FOXA1 expression. All the above data indicated that lncRNA RGMB-AS1 inhibited the progression of NPC by regulating FOXA1. The results of this study were helpful to further understand the mechanism of NPC metastasis, and provided a new theoretical basis for the prevention and treatment of NPC metastasis, which was expected to be better applied in clinical applications. However, in the near future, more studies are needed to further clarify the true role and function of lncRNA RGMB-AS1 as a prognostic marker in NPC cancer biology, and it may become a necessary condition for the development of new therapies for NPC.

## 5 Conclusions

In this study, we reported that a novel lncRNA RGMB-AS1 was down-regulated in NPC. In addition to inhibiting cell viability, proliferation and migration, the overexpression of lncRNA RGMB-AS1 in NPC cells significantly inhibited the EMT process. In addition, the tumorigenic ability of lncRNA RGMB-AS1 was also reduced in *vivo*. We further demonstrated that the tumor suppressive effect of lncRNA RGMB-AS1 can be mediated through direct binding with FOXA1, which added a new perspective to the role of inhibition of NPC occurrence, and thus provided a potential target for the diagnosis, treatment and prognosis of NPC.

## Supplementary Material

Supplemental Material

## Data Availability

The datasets used or/and analyzed during the current study are available from the corresponding author on reasonable request.

## Research highlights

1. The down-regulation of LncRNA RGMB-AS1 expression may be one of the important factors affecting the malignant biological behavior of NPC.

2. LncRNA RGMB-AS1 inhibits NPC proliferation, migration, epithelial mesenchymal transformation and tumorigenesis by regulating FOXA1 in *vivo* and in *vitro*.

3. LncRNA RGMB-AS1 may be an important indicator of clinical prognosis.

## Abbreviations

Long non-coding RNA: lncRNA; RGMB antisense RNA 1: RGMB-AS1; Nasopharyngeal carcinoma: NPC; Forkhead box A1: FOXA1; EMT: epithelial mesenchymal transformation; Quantificational real-time polymerase chain reaction: RT-qPCR; Complementary deoxyribonucleic acid: cDNA; Small interfering RNA: siRNA; RNA immunoprecipitation: RIP.

## Declarations

Ethics committee approval was received for this study from the Ethical Committee of GuiZhou Medical University. The reference number includes number of permit SYXK (黔) 2018-001 and ethical number of animal qualitative qualification 2101395. This clinical trial study has been approved by the Ethics Committee of the Affiliated Hospital of Guizhou Medical University and registered in China Clinical Trial Center (ChiCTR-IPR-14005772). All patients signed informed consent before treatment.
