# Supplementary figures and images for: Long non-coding RNA RGMB-AS1 represses nasopharyngeal carcinoma progression via binding to forkhead box A1
Source: Bioengineered. 2022 Feb 19;13(3):5564–80. doi: 10.1080/21655979.2022.2039495 (PMC8973592; doi:10.1080/21655979.2022.2039495)

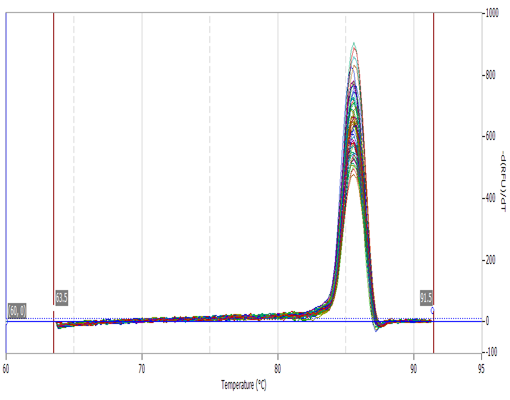

Supplement: Supplemental Material [file KBIE_A_2039495_SM7783.zip › supplementary/actin.png]

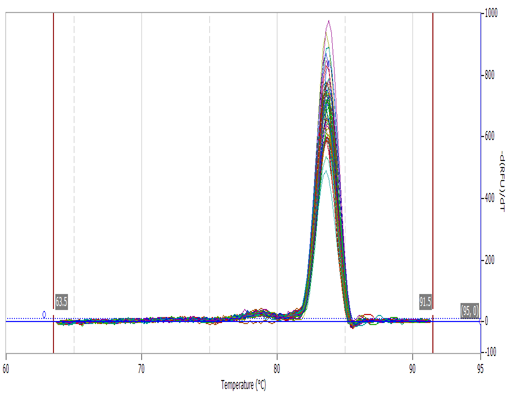

Supplement: Supplemental Material [file KBIE_A_2039495_SM7783.zip › supplementary/FOXA1.png]

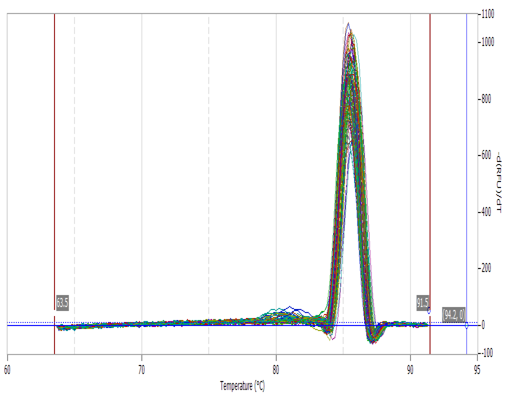

Supplement: Supplemental Material [file KBIE_A_2039495_SM7783.zip › supplementary/lncRNA RGMB AS1.png]
